# Baseline Procalcitonin and C-Reactive Protein Levels in Asymptomatic Individuals From West Africa With and Without *P. falciparum* Parasitemia

**DOI:** 10.1093/ofid/ofag078

**Published:** 2026-02-23

**Authors:** Ricardo Strauss, Solomon T Wafula, Robin Kobbe, Eva Lorenz, Oumou Maiga Ascofaré, Doris Winter, Anthony Afum-Adjei Awuah, John H Amuasi, Boubacar Coulibaly, Ali Sié, Felix Eckelt, Jürgen May, Nicole S Struck

**Affiliations:** Department of Infectious Disease Epidemiology, Bernhard Nocht Institute for Tropical Medicine, Hamburg, Germany; Department of Infectious Disease Epidemiology, Bernhard Nocht Institute for Tropical Medicine, Hamburg, Germany; Department of Disease Control and Environmental Health, School of Public Health, Makerere University, Kampala, Uganda; Department of Infectious Disease Epidemiology, Bernhard Nocht Institute for Tropical Medicine, Hamburg, Germany; German Centre for Infection Research (DZIF), partner site Hamburg-Lübeck-Borstel-Riems, Braunschweig, Germany; Institute for Infection Research and Vaccine Development (IIRVD), University Medical Center Hamburg-Eppendorf, Hamburg, Germany; Department of Infectious Disease Epidemiology, Bernhard Nocht Institute for Tropical Medicine, Hamburg, Germany; German Centre for Infection Research (DZIF), partner site Hamburg-Lübeck-Borstel-Riems, Braunschweig, Germany; Department of Infectious Disease Epidemiology, Bernhard Nocht Institute for Tropical Medicine, Hamburg, Germany; German Centre for Infection Research (DZIF), partner site Hamburg-Lübeck-Borstel-Riems, Braunschweig, Germany; Kumasi Centre for Collaborative Research in Tropical Medicine, Kwame Nkrumah University of Science and Technology, Kumasi, Ghana; Department of Infectious Disease Epidemiology, Bernhard Nocht Institute for Tropical Medicine, Hamburg, Germany; Kumasi Centre for Collaborative Research in Tropical Medicine, Kwame Nkrumah University of Science and Technology, Kumasi, Ghana; Department of Molecular Medicine, Kwame Nkrumah University of Science and Technology, Kumasi, Ghana; Kumasi Centre for Collaborative Research in Tropical Medicine, Kwame Nkrumah University of Science and Technology, Kumasi, Ghana; Department of Global and International Health, Kwame Nkrumah University of Science and Technology, Kumasi, Ghana; Research Group Global and One Health Research, Bernhard Nocht Institute for Tropical Medicine, Hamburg, Germany; German Center for Infection Research (DZIF), partner site Heidelberg, Heidelberg, Germany; Centre de Recherche en Santé de Nouna, Biological Analysis Laboratory, Nouna, Burkina Faso; German Center for Infection Research (DZIF), partner site Heidelberg, Heidelberg, Germany; Centre de Recherche en Santé de Nouna, Biological Analysis Laboratory, Nouna, Burkina Faso; Heidelberg Institute of Global Health (HIGH), Heidelberg University Hospital, Heidelberg University, Heidelberg, Germany; Institute of Clinical Chemistry and Laboratory Medicine, University Medical Center Hamburg-Eppendorf (UKE), Hamburg, Germany; Department of Infectious Disease Epidemiology, Bernhard Nocht Institute for Tropical Medicine, Hamburg, Germany; German Centre for Infection Research (DZIF), partner site Hamburg-Lübeck-Borstel-Riems, Braunschweig, Germany; Tropical Medicine II, University Medical Centre Hamburg-Eppendorf (UKE), Hamburg, Germany; Department of Infectious Disease Epidemiology, Bernhard Nocht Institute for Tropical Medicine, Hamburg, Germany; German Centre for Infection Research (DZIF), partner site Hamburg-Lübeck-Borstel-Riems, Braunschweig, Germany

**Keywords:** procalcitonin, c-reactive protein, malaria, West-Africa

## Abstract

We assessed baseline C-reactive protein (CRP) and procalcitonin levels in asymptomatic individuals from malaria-endemic West Africa. C-reactive protein remained unaffected by *Plasmodium falciparum* parasitemia, while procalcitonin (PCT) was more frequently detectable among malaria-positive individuals. These findings support that CRP thresholds remain valid and highlight the need to explore parasite density–PCT associations.

Procalcitonin (PCT) and C-reactive protein (CRP) are widely used inflammatory host-response biomarkers (IHRB) for diagnosing infections and guiding antimicrobial therapy [1, 2]. Current biomarker cutoffs are primarily derived from populations in high-income countries, potentially limiting their reliability in low-resource settings where comorbidities are common. In malaria-endemic regions, repeated exposure to the malaria parasite Plasmodium falciparum leads to partial clinical immunity, where individuals harbor low-density parasitemia but do not exhibit clinical symptoms [3]. This is due to an acquired immune tolerance, determined by an individual's age and past exposure history, where the immune response controls parasite densities below the pyrogenic threshold [4, 5]. In the absence of fever, this is referred to as “asymptomatic” [3] or “chronic” malaria [5]. Importantly, these infections typically occur at much lower densities than symptomatic cases and may be missed by microscopy (limit of detection estimated at 50–100 parasites/µL under field conditions) [6], but detectible by PCR (limit of detection 0.02 parasites/µL) [7]. Beyond this, persistent exposure to *P. falciparum* can induce polyclonal B-cell activation and inflammatory responses [8, 9]. These effects may result in the production of low-affinity and autoreactive antibodies as well as chronic inflammation, potentially influencing the diagnostic and prognostic value of commonly used biomarkers [8, 10, 11].

Data from malaria-endemic settings suggest that, when interpreted with clinical context, PCT and CRP can guide appropriate antibiotic prescription decisions [12–15]. However, studies assessing these biomarkers' ability to distinguish bacterial from viral infections have largely focused on symptomatic individuals without accounting for malaria endemicity or patients with a malaria coinfection [16]. Previous research demonstrates PCT and CRP levels are substantially higher in bacterial infections than in malarial and viral infections [17]. Meanwhile, the specific effect of parasite positivity on baseline levels of these biomarkers in has not been systematically investigated [18]. Establishing whether this elevates baseline biomarker levels is clinically relevant, as inappropriately high PCT and CRP cutoffs in malaria-endemic regions could lead to missed bacterial diagnoses, while inappropriately low cutoffs might trigger antibiotic overprescription, accelerating antimicrobial resistance development. Although limited data from Sub-Saharan Africa suggests that CRP levels are not substantially influenced by malaria-positivity in asymptomatic individuals [11, 17, 19], more data on IHRBs are needed to consolidate these findings.

Given these knowledge gaps, we aim to investigate how chronic *P. falciparum* parasitemia alters IHRB levels in asymptomatic individuals from Ghana and Burkina Faso. Both countries have a high malaria burden with an estimated 8.14 million recorded cases in Burkina Faso and 6.55 million cases in Ghana in 2023 [20]. If chronic parasitemia consistently elevates baseline IHRB levels in these populations, current lower cutoff values may require upward adjustment to accurately distinguish bacterial infections from chronic malaria. We hypothesize that *P. falciparum*-positivity influences baseline PCT and CRP levels, which is essential for developing context-specific diagnostic and antimicrobial stewardship recommendations in malaria-endemic regions.

## METHODS

### Population, Setting, and Sampling Methods

This study represents a secondary analysis of blood samples collected during a population-based cross-sectional SARS-CoV-2 seroprevalence study conducted in urban residential neighborhoods of Ouagadougou and Bobo-Dioulasso (Burkina Faso), and Accra, Kumasi, and Tamale (Ghana) from 2021 to 2022 [21–23]. Eligible participants were aged ≥10 years without health problems contraindicating blood sample collection or acute symptoms at enrollment. The age inclusion criterion was established by the parent study design based on ethical and practical considerations during the COVID-19 pandemic. Ethical clearance was obtained from the respective national Ethical Board Committees of each participating country.

For the current analysis, we applied specific exclusion criteria: individuals who had COVID-like symptoms within 2 weeks prior to blood collection, those who were SARS-CoV-2 seropositive, and anyone younger than 16 years old. We excluded seropositive SARS-CoV-2 individuals to minimize confounding from recent or past viral infection, as SARS-CoV-2 exposure can elevate CRP and occasionally PCT levels. This restriction allowed us to more precisely isolate the potential effect of *P. falciparum* parasitemia on baseline biomarker levels. Based on a sample size estimation (80% power, alpha 5% and standard deviation of 0.10 for PCT), we determined that 60 individuals per site may be appropriate. We randomly selected participants, stratified by age and sex, from 5 sites: Bobo-Dioulasso and Ouagadougou (Burkina Faso), and Accra, Tamale, and Kumasi (Ghana) for a total sample size of 300.

### Laboratory Procedures

Venous blood samples were collected and cooled until laboratory processing. Ethylenediaminetetraacetic acid (EDTA) plasma was separated from the cell fraction and frozen at −80°C. The red blood cell pellet was resuspended in 8M urea and stored at room temperature until DNA extraction and PCR analysis. The assay for malaria detection was based on Hoffmann et al [24] targeting the high-copy var gene acidic terminal sequence of *P. falciparum* (varATS) using a HotStarTaq Mastermix (Qiagen) on a LightCycler 480II (Roche). For all samples that tested positive in the varATS screening, the parasite density was quantified using a qPCR assay adapted from the method described by Rockett et al [25]. This assay is a duplex real-time qPCR that uses primers and a hydrolysis probe targeting the *Plasmodium falciparum*–specific 18S rRNA gene, optimized for the estimation of parasite load in blood samples. The assay additionally includes the Phocid herpesvirus 1 gene as an internal control to monitor inhibition of the real-time qPCR [26] A qPCR standard curve was prepared using synchronous ring-stage *P. falciparum* (3D7 strain) parasites diluted in *Plasmodium*-negative whole blood. This enables accurate quantification of parasite density in patient samples by comparing to a reference standard with known parasite concentrations [25].

To prepare the reference standard, a synchronized ring-stage cell culture was grown to approximately 8% parasitaemia [27, 28]. An aliquot was stained with SYBR Green nucleic acid gel stain (S9430-.5ML, Sigma Aldrich) according to the manufacturer's instructions and quantified by flow cytometry (Novo-Cyte 3000VYB). The highest standard concentration was then prepared to contain 1.5 × 10^6^ infected red blood cells in a total volume of 300 µL, followed by a 1:10 serial dilution series to generate parasite densities ranging from 1.5 × 10^6^ to 1.5 × 10^1 ^parasites/µL. DNA was extracted from 200 µL aliquots of standard dilutions using the same protocol as patient samples [29], ensuring identical processing. Parasite density in each patient sample was calculated as parasites/µL by comparing cycle threshold (Ct) values to the standard curve with defined parasite concentrations.

The biomarkers CRP and PCT were measured in ETDA plasma in CE and IVD certified assays at the ISO 15089 accredited Institute for Clinical Chemistry and Laboratory Medicine, UKE. C-reactive protein was measured using the Atellica CH CRP 2 assay, which uses polystyrene latex particles coated with anti-CRP antibodies. The coefficient of variation (CV) for the turbidity method was 2.14% for level 1 at 963 mg/L. The CRP assay has a measurement range of 4 to 304 mg/L. The PCT was measured using the Atellica IM BRAHMS Procalcitonin system (Siemens Atellica Solution, Germany, Erlangen). The PCT assay is a 2-site sandwich immunoassay using direct chemiluminescence (CLIA) that utilizes an acridinium ester-labeled mouse monoclonal anti-PCT antibody and 2 fluorescein-labeled mouse monoclonal anti-PCT antibodies. The CV for the CLIA method was 5.76% for level 1 at 0.42 µg/L. The PCT assay has a measurement range of 0.02–50 µg/L.

### Data Analysis

Descriptive statistics, including median and interquartile range, were used for numerical data. For results below the lower limit of quantification (LoQ), we applied an imputation method using half the LoQ value. After imputing values below the LoQ, median and interquartile range were calculated. Frequencies and proportions were calculated for categorical data. Infographics were used to illustrate the distribution of PCT and CRP by age-sex strata, and *P. falciparum* status. A simple logistic regression for the association between the presence or absence of malaria parasites and the possibility of detectable values of PCT and CRP values was performed.

## RESULTS

### Participant Characteristics and Malaria Status

Of the 300 participants (median age = 33.0 years, IQR = 24.0, 50.0), 161 (53.6%) were female, 180 (60%) individuals from Ghana and 120 (40%) from Burkina Faso, 47 (15.7%) tested positive for *P. falciparum* infection (Supplementary Table 1). We performed qPCR to determine parasite densities among the 47 malaria-positive individuals, which ranged from below 0 to 74.92 parasites/µL (median 0.2109 parasites/µL [IQR 0.1339–2.2033]). Notably, all parasite densities fell below the WHO threshold for “low-density parasitaemia” [30]. Malaria microscopy was not performed.

### Distribution of C-Reactive Protein and Procalcitonin

A total of 246 samples (82.0%) had CRP values below the LoQ (4.00 mg/L), with a maximum of 209.34 mg/L. Meanwhile, a total of 253 samples (84.3%) had PCT values below the LoQ (0.06 µg/L), with a maximum of 1.15 µg/L.

Median PCT levels were identical (0.01 µg/L) in both *P. falciparum*-positive and -negative participants, with a wider spread and a few elevated values in the malaria-positive group. However, the clinical relevance of this difference is limited given the similar central tendency of values. C-reactive protein levels did not show evidence for a difference between groups (median = 2 mg/L in both).

We found no significant association between *P. falciparum* infection and having detectable CRP levels (inverse OR = 1.30, 95% CI: .57–2.70). However, *P. falciparum* infection was significantly associated with increased odds of detectable PCT levels, with an odds ratio of 2.44 (95% CI: 1.15–5.00), indicating infected individuals were more than 2 times more likely to have detectable PCT compared with noninfected individuals (Table 1). We examined the relationship between parasite density and PCT or CRP levels but did not observe any significant associations (Supplementary Figures 1-4).

**Table 1.** Association Between *P. falciparum* Parasites and Detectable CRP/PCT Levels

	CRP, n (%)		Unadjusted OR (95% CI)	PCT, n (%)		Unadjusted OR (95% CI)
	Detectable	Nondetectable		Detectable	Nondetectable	
*P. falciparum* parasites	…	…	…	…	…	…
Absent	44 (17.4)	209 (82.6)	Ref	34 (13.4)	219 (86.6)	Ref
Present	10 (21.3)	37 (78.7)	1.30 (.57–2.70)	13 (27.7)	34 (72.3)	2.44 (1.15–5.00)

Abbreviations: CI, confidence interval; CRP, C-reactive protein; n, sample size; OR, odds ratio; PCT, procalcitonin; Ref, reference.

## DISCUSSION

To our knowledge, this is the first study to assess baseline CRP and PCT levels in a randomly selected, asymptomatic population from 2 malaria-holoendemic West African countries. We found consistent CRP levels across age, sex, and malaria status. Moreover, the likelihood of CRP detectability did not significantly differ between *P. falciparum*-positive and -negative individuals. This suggests that, within the limitations of our study, low-grade parasitemia does not alter baseline CRP levels in otherwise asymptomatic adults. Thus, CRP lower cut-off currently in use might not need adjustment in adult population living in malaria-endemic settings. In contrast, parasitemia was significantly associated with detectable PCT levels; however, given the substantial proportion of measurements below the LoD and the exploratory nature of our analysis, this finding should be interpreted cautiously. We cannot determine whether this reflects a true biological response to subclinical malaria-related inflammation or is partly driven by methodological constraints. Further research could track PCT changes over time in the same individuals and establish dose-response relationships between parasite density and biomarker levels. Age groups across varying levels of malaria endemicity might exhibit different inflammatory profiles, potentially requiring regional calibration for biomarker thresholds.

Further, studies involving individuals with confirmed single-pathogen infections—bacterial or viral, with and without malaria coinfection—is needed to validate IHRB thresholds for antimicrobial guidance in endemic contexts.

While malaria parasite-positivity contributes to low-grade inflammation [9], our findings suggest this effect may not substantially alter baseline CRP levels at the population level in areas with stable transmission. This aligns with evidence from West Africa showing that CRP levels remain consistent in malaria-endemic populations among individuals with chronic parasitemia [11, 19]. However, some evidence suggests a more nuanced relationship with parasite density. A study from Ghana found elevated CRP levels specifically in otherwise asymptomatic adults with high parasitemia, suggesting that activation of immune processes responsible for triggering CRP production depends on parasite density [19, 31]. Similarly, a study from South-East Asia, where *Plasmodium vivax* predominates, found moderate CRP increases correlating with increasing parasite density [32].

Several limitations should be considered. Our study population overrepresented females and young adults, limiting external generalizability to children and older populations. This is particularly important as children may exhibit stronger pro-inflammatory immune responses to malaria compared with semi-immune adults. Importantly, only 15.7% of participants were *P. falciparum*-positive, and malaria status was not accounted for during sampling, which might have affected our ability to detect subtle differences in biomarker levels between groups. Additionally, potential variations in sample handling and storage conditions may have affected biomarker stability. Finally, the parent study design lacked comprehensive assessments of malaria exposure, recent antimalarial treatment or clinical onset of symptoms, limiting our ability to explore these factors in relation to baseline biomarker levels. Future studies should include more representative populations with widespread prior SARS-CoV-2 exposure to assess how overlapping endemic and epidemic infections jointly influence baseline inflammatory biomarker levels.

## Supplementary Material

ofag078_Supplementary_Data

## Supplementary Data

Supplementary materials are available at *Open Forum Infectious Diseases* online. Consisting of data provided by the authors to benefit the reader, the posted materials are not copyedited and are the sole responsibility of the authors, so questions or comments should be addressed to the corresponding author.
