# Supplementary material for: Baseline Procalcitonin and C-Reactive Protein Levels in Asymptomatic Individuals From West Africa With and Without P. falciparum Parasitemia
Source: Open Forum Infect Dis. 2026 Feb 23;13(3):ofag078. doi: 10.1093/ofid/ofag078 (PMC12967068; doi:10.1093/ofid/ofag078)
Supplement: ofag078_Supplementary_Data [file ofag078_supplementary_data.zip › OFID_Supp_Figures_20251216_alttext.docx]

**Supplementary Figures**

**
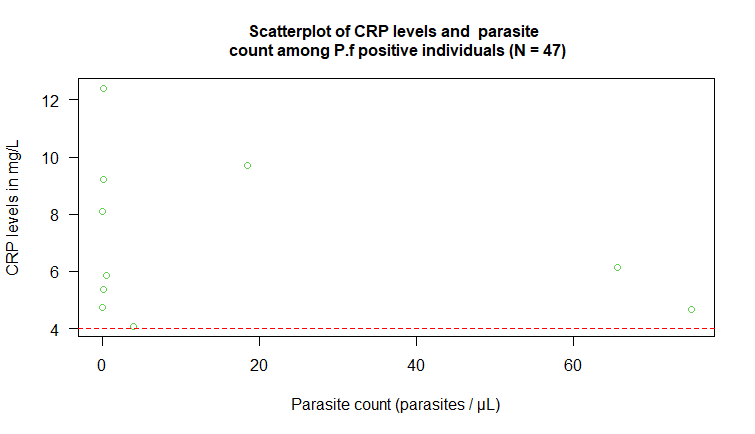
**

Figure S1. Scatterplot showing the relationship between C-reactive protein (CRP) concentrations and parasite density in asymptomatic P. falciparum infections (N=47). The red horizontal line represents the lower clinical cut-off value (4 mg/L). Parasite densities were determined by qPCR and are expressed as parasites/µL.

[Alt text: Scatter plot showing no apparent correlation between parasite density (x-axis, 0-75 parasites/µL) and CRP concentrations (y-axis, 0-12 mg/L), with red horizontal line at 4 mg/L threshold].


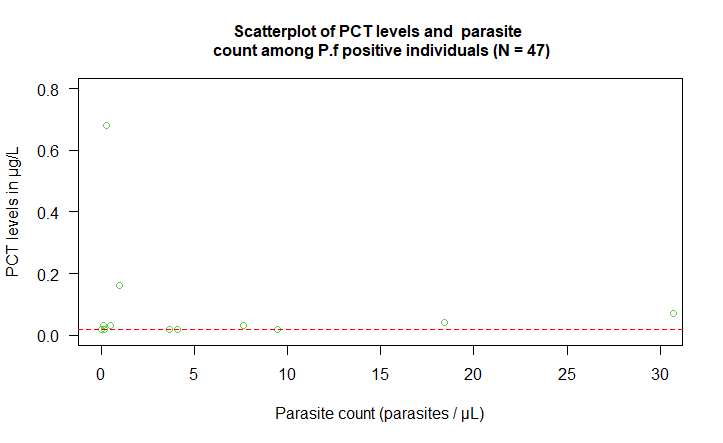


Figure S2. Scatterplot showing the relationship between Procalcitonin (PCT) concentrations and parasite density in asymptomatic P. falciparum infections (N=47). The red horizontal line represents the lower clinical cut-off (0.02 μg/L). Parasite densities were determined by qPCR and are expressed as parasites/µL.

[Alt text: Scatter plot showing no apparent correlation between parasite density (x-axis, 0-75 parasites/µL) and PCT concentrations (y-axis, 0-0.8 μg/L), with red horizontal line at 0.02 μg/L threshold].


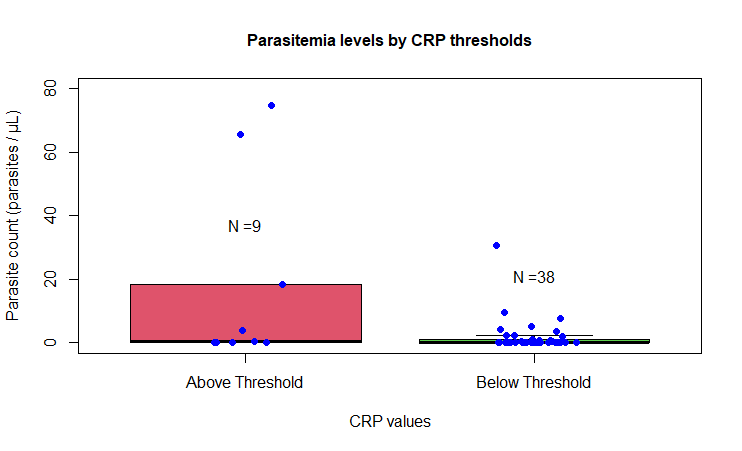


Figure S3. Boxplots comparing the distribution of parasite densities in participants with C-reactive protein (CRP) levels above versus below the lower clinical cut-off (4 mg/L). Boxplots show individual data points and indicate median as well as interquartile range (IQR). Parasite densities were determined by qPCR and are expressed as parasites/µL.

[Alt text: Two box plots showing similar parasite density distributions for participants with CRP above versus below the clinical threshold of 4 mg/L, with overlaid individual data points].


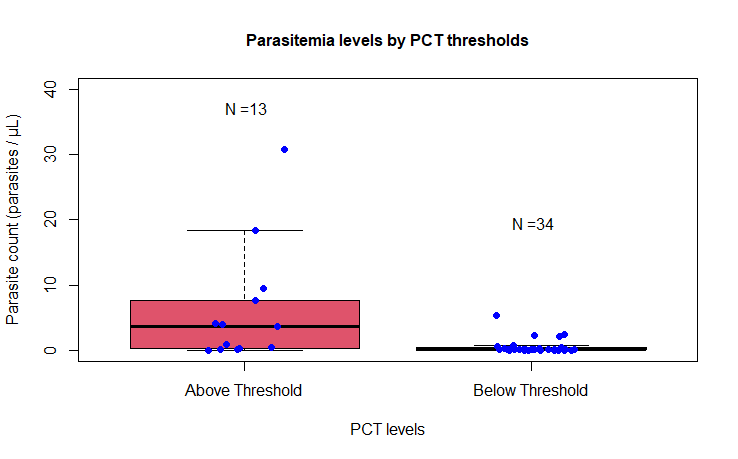
Figure S4. Boxplots comparing the distribution of parasite densities in participants with Procalcitonin (PCT) levels above versus below the lower clinical cut-off (0.02 μg/L). Boxplots show individual data points and indicate median as well as interquartile range (IQR). Parasite densities were determined by qPCR and are expressed as parasites/µL.

[Alt text: Two box plots showing similar parasite density distributions for participants with PCT above versus below the clinical threshold of 0.02 μg/L, with overlaid individual data points].
