# Supplementary material for: Baseline Procalcitonin and C-Reactive Protein Levels in Asymptomatic Individuals From West Africa With and Without P. falciparum Parasitemia
Source: Open Forum Infect Dis. 2026 Feb 23;13(3):ofag078. doi: 10.1093/ofid/ofag078 (PMC12967068; doi:10.1093/ofid/ofag078)
Supplement: ofag078_Supplementary_Data [file ofag078_supplementary_data.zip › OFID_Supp_Table 1_20251218.docx]

***Supplementary Table S1: Demographic characteristics of participants by country and Plasmodium falciparum status.***

| **Characteristics** | **Overall, (N = 300) (%)** | **Plasmodium falciparum status** | | **Country** | |
| --- | --- | --- | --- | --- | --- |
|  |  | ***Positive*** | ***Negative*** | ***Burkina Faso*** | ***Ghana*** |
| **Totals** | 300 (100.0) | 47 (15.7) | 253 (84.3) | 120 (40.0) | 180 (60.0) |
| **Sex** |  |  |  |  |  |
| Female | 161 (53.7) | 19 (11.8) | 142 (88.2) | 65 (40.4) | 96 (59.6) |
| Male | 139 (46.3) | 28 (20.1) | 111(79.9) | 55 (39.6) | 84 (60.4%) |
| **Age in years** |  |  |  |  |  |
| Mean (SD) = 36.6 (16.5) |  |  |  |  |  |
| **Age – Sex Strata** |  |  |  |  |  |
| 16 – 19, Female | 19 (6.3) | 06 (31.6) | 13 (68.4) | 12 (63.2) | 7 (36.8) |
| 16 – 19, Male | 21 (7.0) | 03 (14.3) | 18 (85.7) | 10 (47.6) | 11 (52.4) |
| 20 – 44, female | 99 (33.0) | 09 (9.1) | 90 (90.9) | 42 (47.6) | 57 (57.6) |
| 20 – 44, Male | 76 (25.3) | 14 (18.4) | 62 (81.6) | 32 (42.1) | 44 (57.9) |
| 45+, Female | 43(14.3) | 04 (9.3) | 39 (90.7) | 11 (25.6) | 32 (74.4) |
| 45+, Male | 42 (14.0) | 11 (26.2) | 31 (73.8) | 13 (31.0) | 29 (69.0) |
