# Supplementary figures and images for: Baseline Procalcitonin and C-Reactive Protein Levels in Asymptomatic Individuals From West Africa With and Without P. falciparum Parasitemia
Source: Open Forum Infect Dis. 2026 Feb 23;13(3):ofag078. doi: 10.1093/ofid/ofag078 (PMC12967068; doi:10.1093/ofid/ofag078)

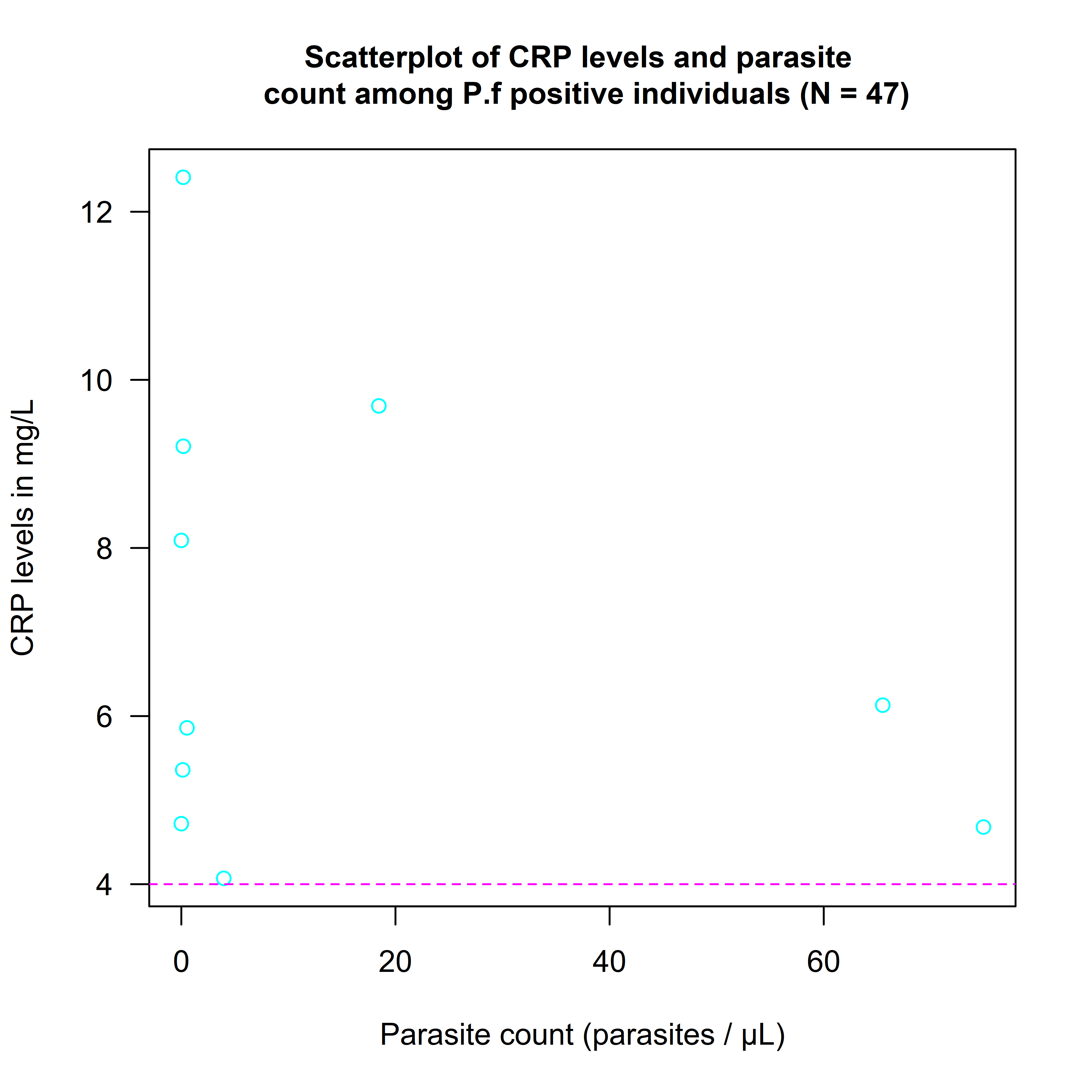

Supplement: ofag078_Supplementary_Data [file ofag078_supplementary_data.zip › Figure S1.tif]

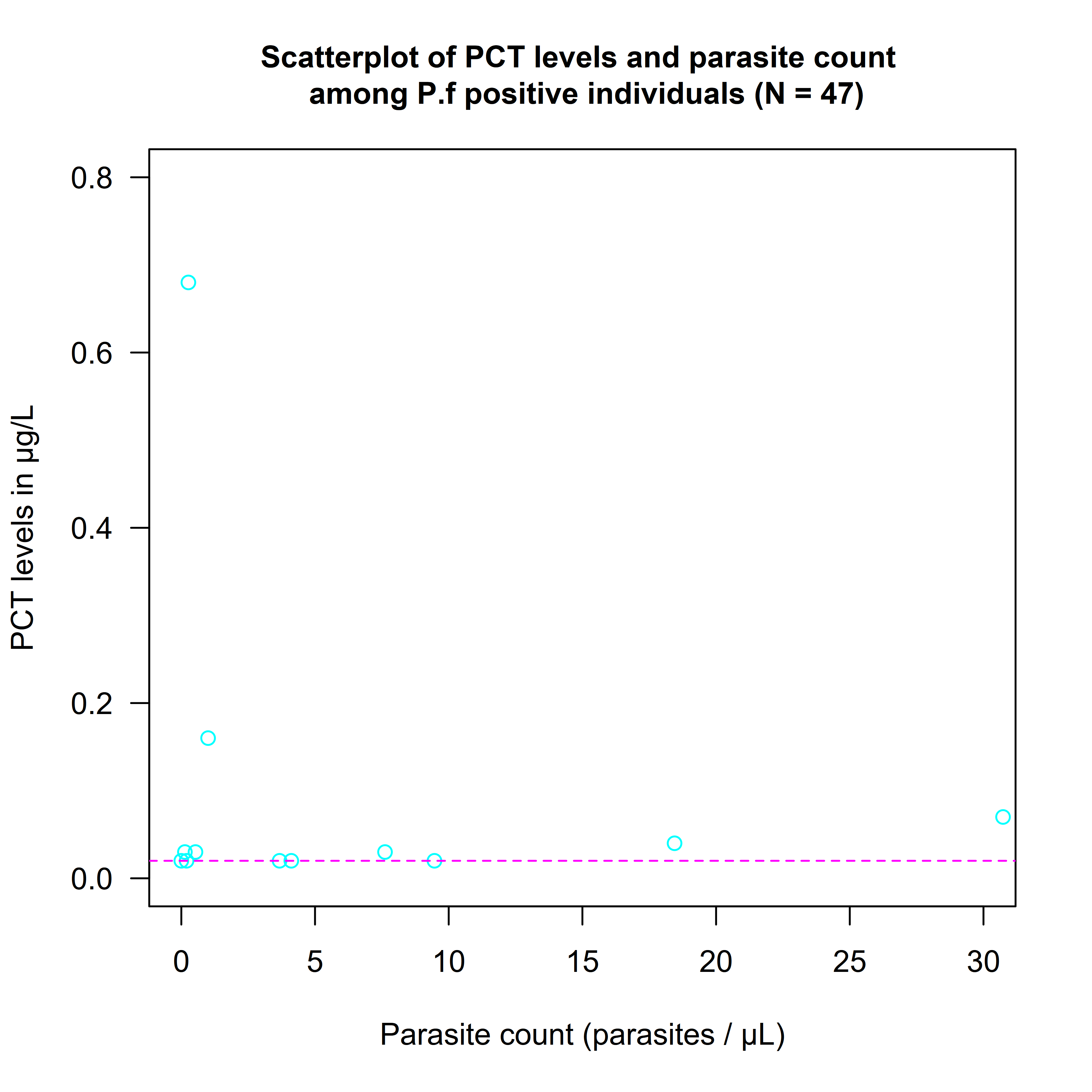

Supplement: ofag078_Supplementary_Data [file ofag078_supplementary_data.zip › Figure S2.tif]

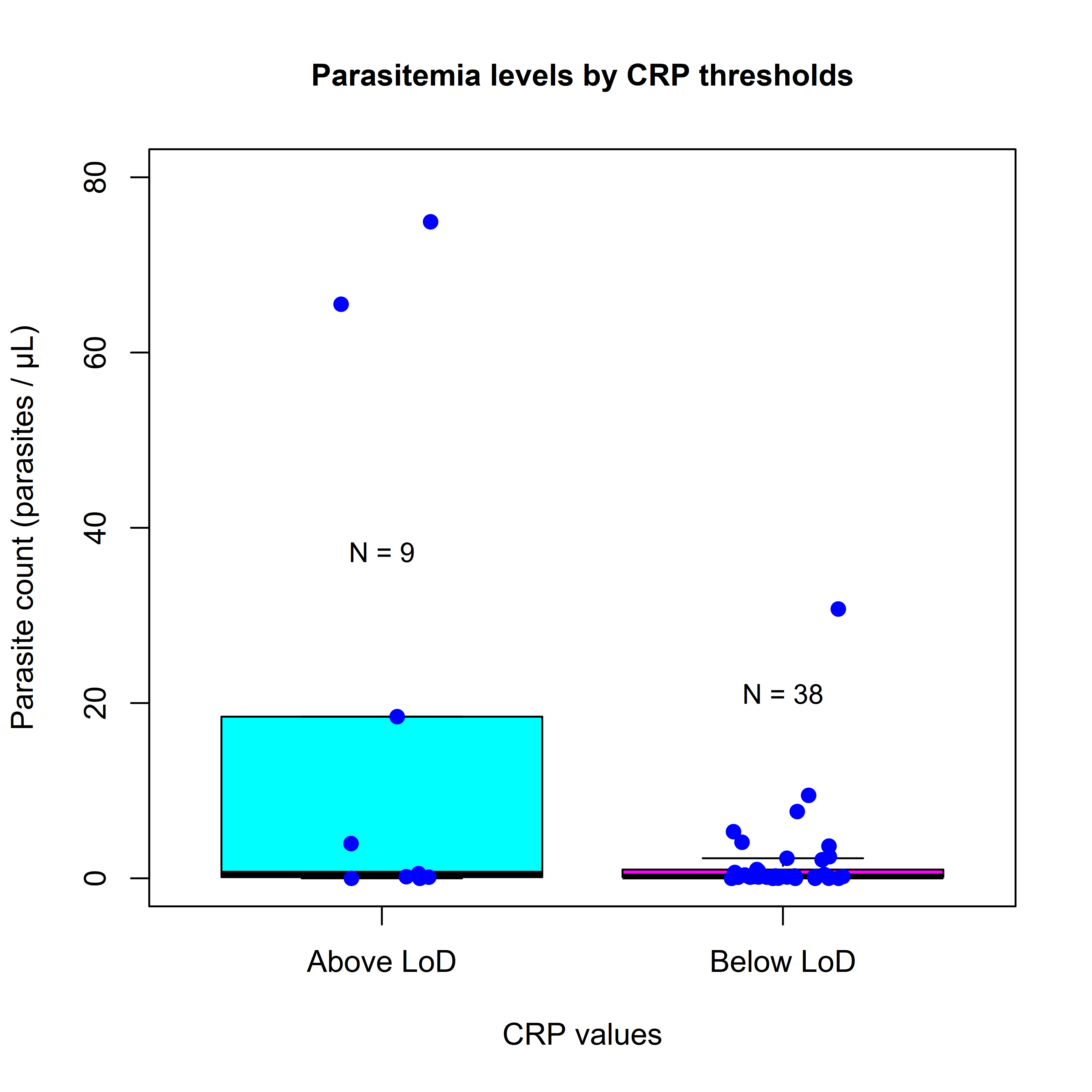

Supplement: ofag078_Supplementary_Data [file ofag078_supplementary_data.zip › Figure S3.tif]

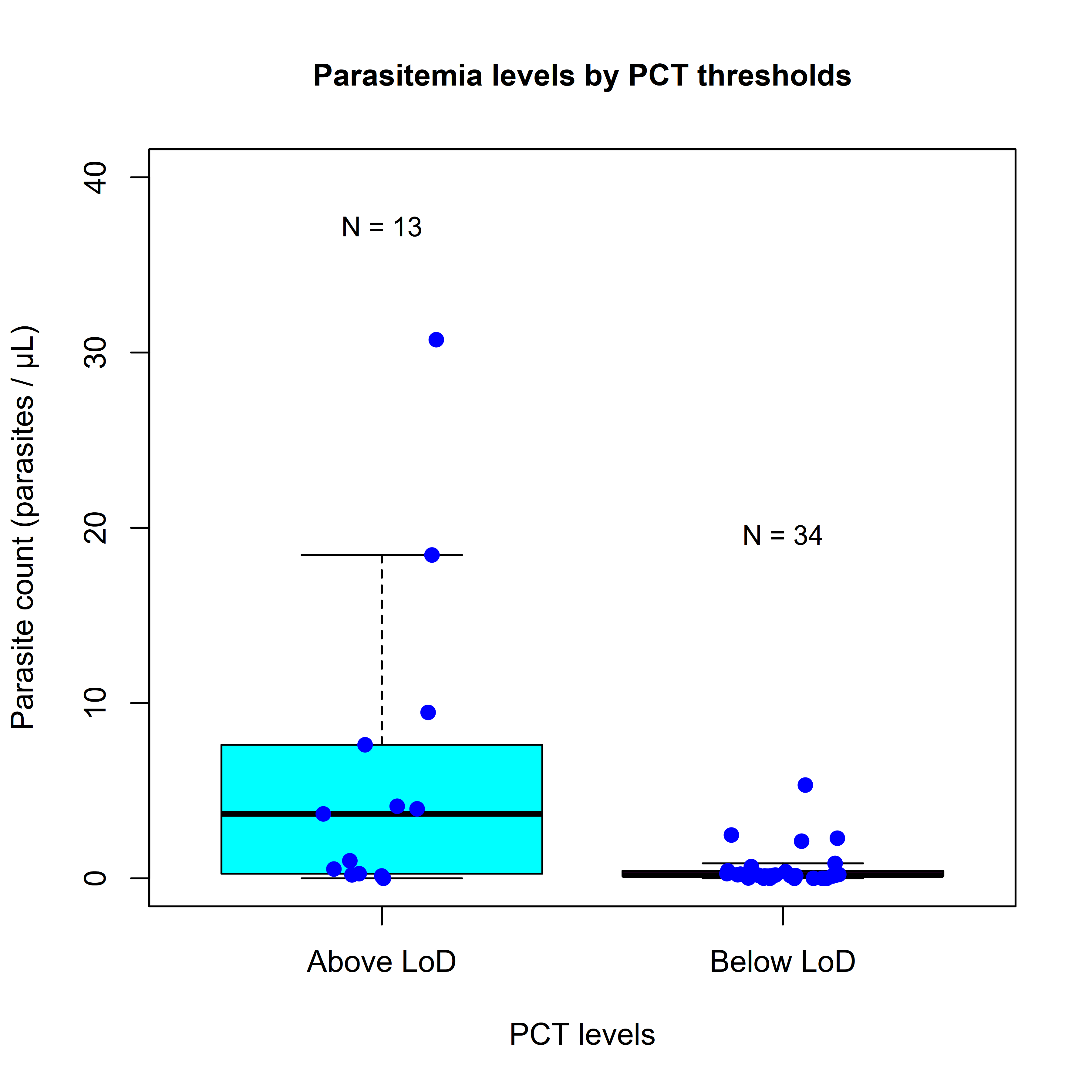

Supplement: ofag078_Supplementary_Data [file ofag078_supplementary_data.zip › Figure S4.tif]
